# A decade of the World Register of Marine Species – General insights and experiences from the Data Management Team: Where are we, what have we learned and how can we continue?

**DOI:** 10.1371/journal.pone.0194599

**Published:** 2018-04-06

**Authors:** Leen Vandepitte, Bart Vanhoorne, Wim Decock, Sofie Vranken, Thomas Lanssens, Stefanie Dekeyzer, Kevin Verfaille, Tammy Horton, Andreas Kroh, Francisco Hernandez, Jan Mees

**Affiliations:** 1 Flanders Marine Institute (VLIZ), Wandelaarkaai 7, Oostende, Belgium; 2 School of Biological Sciences, UWA Oceans Institute, Crawley (Perth), WA, Australia; 3 National Oceanography Centre, Southampton, United Kingdom; 4 Natural History Museum, Vienna, Austria; 5 Ghent University, Ghent, Belgium; Universitetet i Bergen, NORWAY

## Abstract

The World Register of Marine Species (WoRMS) celebrated its 10^th^ anniversary in 2017. WoRMS is a unique database: there is no comparable global database for marine species, which is driven by a large, global expert community, is supported by a Data Management Team and can rely on a permanent host institute, dedicated to keeping WoRMS online. Over the past ten years, the content of WoRMS has grown steadily, and the system currently contains more than 242,000 accepted marine species. WoRMS has not yet reached completeness: approximately 2,000 newly described species per year are added, and editors also enter the remaining missing older names–both accepted and unaccepted–an effort amounting to approximately 20,000 taxon name additions per year. WoRMS is used extensively, through different channels, indicating that it is recognized as a high-quality database on marine species information. It is updated on a daily basis by its Editorial Board, which currently consists of 490 taxonomic and thematic experts located around the world. Owing to its unique qualities, WoRMS has become a partner in many large-scale initiatives including OBIS, LifeWatch and the Catalogue of Life, where it is recognized as a high-quality and reliable source of information for marine taxonomy.

## Introduction

The World Register of Marine Species (WoRMS) was launched online in 2007. An official inauguration followed in June 2008, and this received extensive coverage in the media. The idea to set up WoRMS came from within the Census of Marine Life (CoML) community, where marine scientists wanted to create a world database of ocean organisms (e.g.[1]). At the time of the official launch, WoRMS contained 122,500 validated marine species names and an initial consortium of 55 researchers from 17 countries planned for its completion by 2010, when the First Census of Marine Life would be released [2,3].

The initial goal of WoRMS has not changed over the past 10 years: the Register still aims “*to provide freely online the most authoritative list of names of all marine species ever published*”. Based on available numbers at the start of WoRMS, it was envisioned that the then estimated 230.000 described marine species could be incorporated into the database by 2010. It took a few years longer: this first target was reached on 15 July 2015. At roughly the same time, scientists recognized that (1) the original estimate was an underestimation, (2) that WoRMS also needed to keep up with the newly described species and (3) that also all the older published taxon names–i.e. synonyms that are no longer or only sporadically used—should be included.

WoRMS is hosted at the Flanders Marine Institute (VLIZ), in Oostende (Belgium). VLIZ has committed to keeping WoRMS online and available to its many users. A Data Management Team (DMT) at VLIZ provides support–both technical and content-wise–to the editorial board and the user community. The DMT consists of both technical and scientific staff, each following up on specific aspects of WoRMS. Although VLIZ was only founded in 1999 and is a rather small-scale marine institute, it has built well-renowned expertise in the field of hosting and managing large databases related to marine biodiversity.

The number of staff in the DMT can vary over time, depending on available funding. Currently, the DMT is largely funded through core funding of the Flanders Marine Institute (VLIZ) and LifeWatch Belgium, which is part of the European E-Science Infrastructure for Biodiversity and Ecosystem Research. LifeWatch is a distributed virtual laboratory, which is used for different aspects of biodiversity research. The World Register of Marine Species is an important component of the Taxonomic Backbone of LifeWatch, which aims to bring together taxonomic and species-related data to fill the gaps in our knowledge.

## WoRMS governance

Since the beginning, WoRMS has been managed at three different levels: (1) the Steering Committee (SC), (2) the Editorial Board, and (3) the Data Management Team (DMT), each having their own tasks and priorities.

The elected WoRMS Steering Committee (SC) represents the members of the WoRMS Editorial Board in all matters that relate to the databases, including acting as a liaison with other international projects and initiatives. It takes care of the day-to-day business of WoRMS and sets priorities for future activities. The SC acts in close collaboration with the DMT, which has an ex-officio membership in the SC. The SC carries responsibility for the scientific accuracy of the database, although it is recognized that no database of such a size is without errors and omissions. Members of the SC also evaluate the database download requests, ensuring proper use of the data and negotiating exceptional uses outside the standard license.

At the start of WoRMS in 2007, the SC consisted of 14 members. In 2009, the composition of the SC was formalized: a rotation system of 12 elected members was proposed and a number of ex-officio members–representing regional and thematic registers–were allowed. In 2015, some changes to the statutes were made: from then on, each member could serve for a period of 3 years–with the possibility of re-election–and should be nominated/elected by the Editorial Board. Regional and thematic representatives no longer held an ex-officio position, but also needed to be elected by the editorial board in order to sit on the SC, only leaving a representative of the DMT as ex-officio member in the SC. The DMT is responsible for the organization of the nominations and–if there are more candidates than available places–elections. A Chair and Vice-chair are elected within the SC, and each of them serves for a period of 3 years, again with the possibility of re-election.

The SC meets twice a year, to discuss matters at hand and to plan for the future of WoRMS. If urgent matters arise in between meetings, these are taken up through email. All discussions, conclusions, and action points are communicated to the Editorial Board and are made available online, thereby establishing a very open communication with the broader user community, and allowing for the greatest possible transparency in WoRMS-related matters.

The WoRMS Editorial Board includes all active editors and data providers. Their main task is to take responsibility for one or more taxonomic groups, themes or regions under their expertise, by adding newly published taxa to WoRMS, correcting errors and constantly being on the look-out for information that might be of value to WoRMS. The editor community is the pivot of WoRMS: they dedicate time to making WoRMS more complete and helping the DMT in answering user questions and fixing issues that arise from standard quality control procedures. Without the editors, WoRMS could not be where it is today.

The Editorial Board is a very dynamic body (see also section ‘Editors – the driving force behind WoRMS‘): new editors regularly come on board, to help out already active editors in lessening their workload and bringing in particular expertise. But editors also retire from their WoRMS tasks, and replacement needs to be sought. Retirement from WoRMS is mostly related to either a change in jobs–and no longer having the time to work on WoRMS–or coincides with retirement from their day-job. Retiring editors are asked to help look for a proper editorial replacement, so the maintenance of a taxon group can be guaranteed over time. In odd cases–e.g. with the passing away of an editor–the DMT and SC need to consult the Editorial Board and ask them to help look for an expert to fill the vacant place. A distinction is made between taxonomic editors–with rights to make additions and changes to all modules in the databases–and thematic editors, who edit and add information on all modules except taxonomy. The latter includes editors that work on both the regional and thematic species databases.

The WoRMS Data Management Team (DMT) has been in place since the very start of WoRMS in 2007, and was already as early as 2003, taking care of the preceding sub-registers of WoRMS. Although its composition has changed over time, their dedication and tasks have remained the same. The DMT is responsible for keeping the database online, protecting its integrity and the persistence of the unique identifiers (AphiaIDs). The DMT makes monthly archives of the database, in a secure facility, allowing to revert to a previous version if needed. The DMT is also the first line of support for the WoRMS users. If the DMT are unable to fix or solve something and the problem requires taxonomic (or thematic/regional) expertise, then the editors are contacted. The DMT supervises all ongoing editing activities and supports editors where needed in their online work. They also bulk-upload large amounts of data (taxonomy, distributions, literature or traits) and maintain the taxonomy upon request of the editors. For orphan groups within WoRMS–where there is no responsible editor–the DMT takes up the editorial responsibilities, until an expert steps in. The organization of meetings, workshops, and nomination and election rounds for the SC also falls under their responsibility.

## Global, regional and thematic species databases

While WoRMS is the home of all extant, marine species, a number of editors wanted to document all known species within their own taxonomic group of expertise in a single database, requiring addition of non-marine and/or fossil representatives. To accommodate this, the DMT developed the possibility to create separate portals, with a focus on either a taxonomic group (Global Species Databases–GSDs), a theme or a region (Thematic and Regional Species Databases–TSDs and RSDs). This not only helps the involved editors in the management of their group, but also offers extra visibility, recognition of, and attribution to their work, as each portal has an individual citation and–if desired–a DOI. In addition, the individual portals give editors the opportunity to focus on the specifics of their group, e.g. by providing more background information than is possible through the global WoRMS portal. We refer to [4] on how these registers are created and maintained on a database level and to [5] on how data entry consistency is maintained across all these registers.

**Fig 1:**
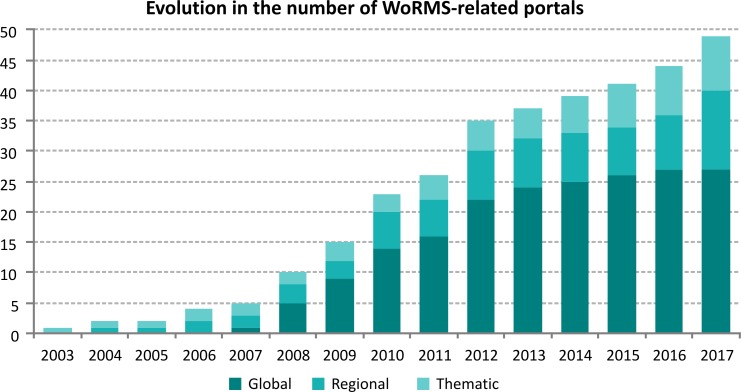
Evolution in the number of WoRMS-related portals, sub-categorized in global, regional and thematic species databases, represented cumulatively.

Over the years, the number of available online portals has grown steadily. Only exceptionally has a portal has been taken offline, as it became part of another portal. So far, 49 separate online portals have been created, with a focus on GSDs, dedicated to a specific taxonomic group (Fig 1). The registers pre-dating the launch of WoRMS in 2007 all deal with marine species within a more confined geographical area (e.g. Belgian [BeRMS] and European marine waters [ERMS]) and were created during several projects). These early regional registers contributed to the initiation of WoRMS in 2007, but have kept their own identity through dedicated portals (see also [4].

## Editors–the driving force behind WoRMS

**Fig 2:**
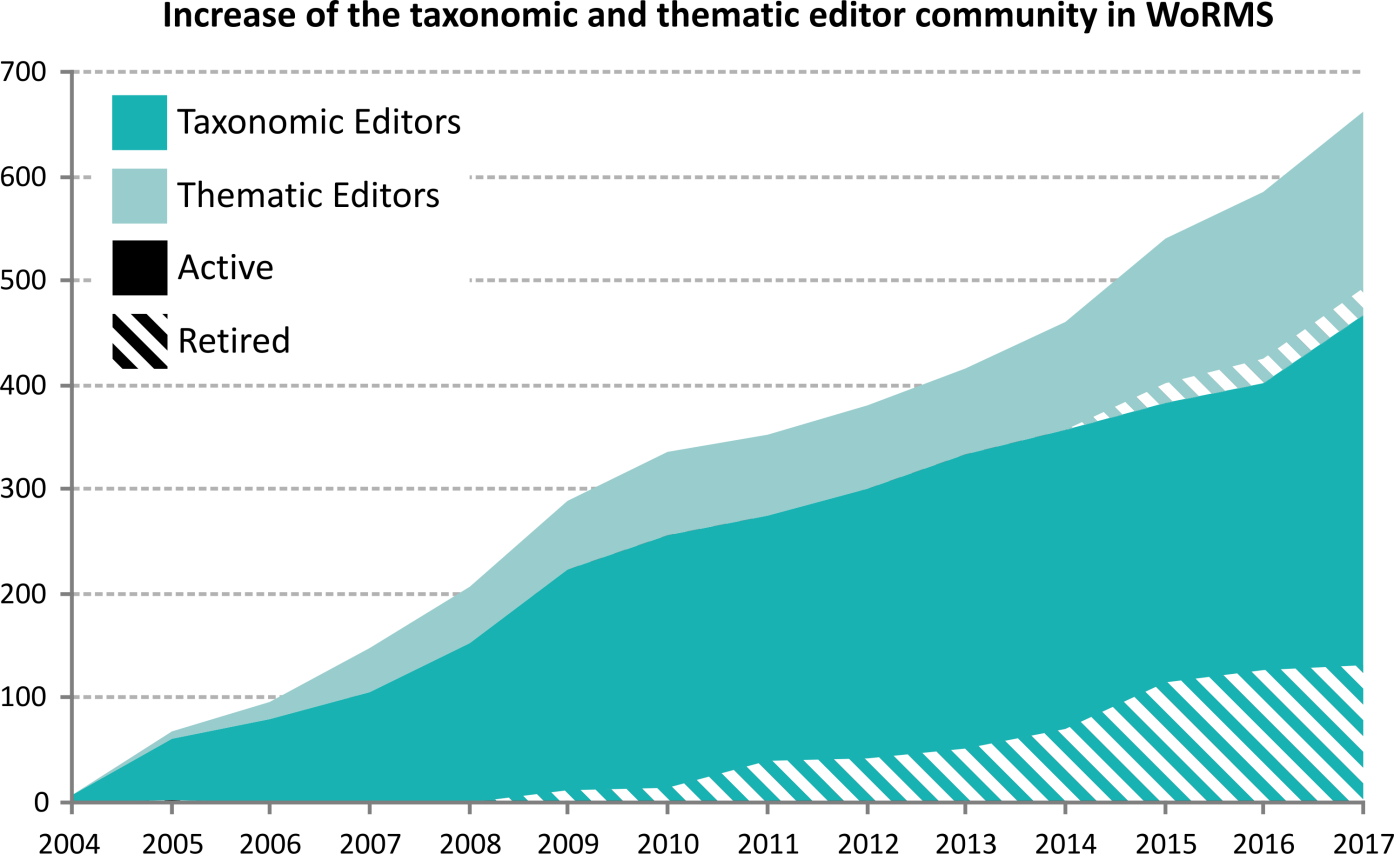
Growth in the number of taxonomic and thematic editors involved in WoRMS. For both groups, editors have retired over the years, but a steady increase for both groups is being maintained.

Similar to the launch of new Global & Thematic Species Databases, the numbers of involved WoRMS taxonomic and thematic editors has also evolved steadily over the last ten years, and some editors were present even before the start of WoRMS in 2007 (Fig 2).

The number of editors helping to maintain and update WoRMS varies due to e.g. retirement or ceasing of editor activities owing to lack of time. Although many editors retired from their WoRMS tasks in the years 2011 and 2013–2015 (Fig 2), WoRMS has been able to keep a steady growth of its editorial board, partly linked to the extra registers that are linked to WoRMS. A similar trend is visible for thematic editors, with largest growth since 2013. Since this time, more thematic and regional registers have been brought online, each time involving on average 16 new thematic editors. The number of thematic editors is in fact an underestimation, as many taxonomic editors are also doing thematic data editing, which is not taken into account in Fig 2. There are currently 337 taxonomic editors and 169 thematic editors active on WoRMS. Editors are spread over 57 countries worldwide and represent 325 institutes.

Over the last 5 years, there is a clear trend in moving towards more editors per taxonomic group, thus dividing the workload and allowing everyone to invest time in their specific group of expertise within a larger group (e.g. a family or order within a class or phylum). This trend is strongly supported by the WoRMS Steering Committee and Data Management Team, as more hands make lighter work. In cases of large editor groups, there is one or a few chief taxonomic experts, who oversee the group as a whole, take responsibility in case of questions or conflict situations, and follow up on the activity of the other involved editors. Within such an editor group, each involved expert either has responsibility over a number of genera, families or orders, or there is an overall agreement that everyone edits all taxa within the group. Both approaches are being used within WoRMS and have proven to be equally successful. In many cases, the chief taxonomic editors not only look at the expertise of the people they involve, but also at their geographical distribution, thereby aiming for a global coverage. Some examples of taxonomic groups that evolved from a single- to multiple-effort approach include Amphipoda and diatoms, respectively going from 5 to 33 editors and from 1 to 19 editors. The expansion of these two editorial networks was closely linked with the organization of WoRMS-LifeWatch taxonomic editor workshops, allowing all experts to meet in person, to learn to work using the online editing interface, to discuss short- and long-term plans for their register, and to discuss the launch of new GSD portals. Whereas the taxonomic sub-sets of WoRMS are gradually evolving from a one to many involved experts, the thematic and regional sub-sets generally start off with a larger editorial network, seemingly characterized by the nature of these registers as they mostly cover a substantial part of the marine life and are not bound by taxonomic boundaries. This includes e.g. the thematic registers of introduced [6] and cave species [7] and the regional registers of Antarctica [8] and China [9].

**Fig 3:**
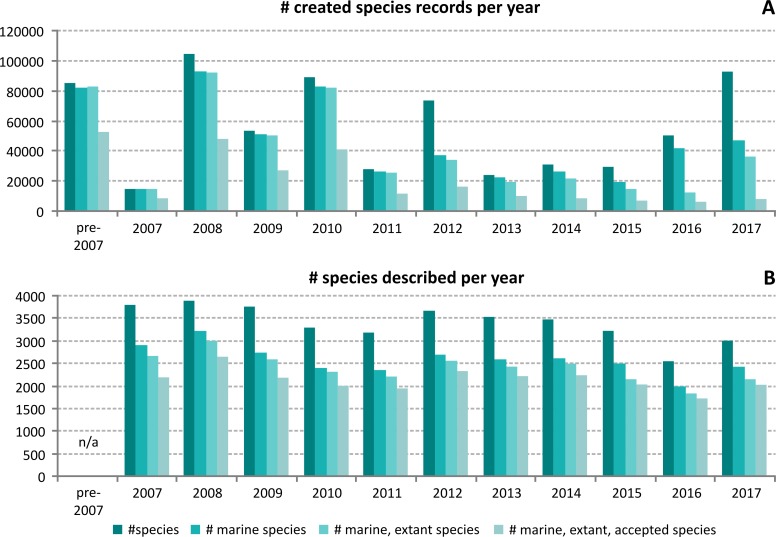
(A): Overview of the number of species records created in WoRMS per year over the last ten years. A distinction is made between all accepted & unaccepted species across all environments and including fossils (#species), all accepted & unaccepted marine species and including fossils (#marine species), all accepted & unaccepted marine species excluding purely fossil species (#marine, extant species) and the number of marine, extant, accepted species which are the core focus of WoRMS. (B): Overview of the number of species described per year and documented in the World Register of Marine Species (WoRMS).

The number of species records that are created annually in the WoRMS database is highly variable (Fig 3A). Strong peaks appear in 2008 and 2010, which correspond to the additions of species in the start-up of WoRMS (2008) and the completion of the First Census of Marine Life (2010) [10]. The peak in 2012 relates to the synchronization with AlgaeBase [11], where many new species names were introduced into the database, the majority being non-marine species. In 2017, a similar situation appears through two major data imports for the diatoms and the Diplopoda. Overall, the number of species name records being added annually through WoRMS is at least 20,000 per year (including synonyms)–with an annual average of more than 56,000 over the last 10 years–indicating that there are still a lot of species names missing. If we compare the number of added species records per year to the number of species being described per year, the relation is on average 4:1, and is not yet levelling off (Fig 3A vs. Fig 3B). This clearly indicates that the work of the editorial board is not limited to just keeping track of newly published names, but still has a strong focus on completing the register with already existing species names. On average, yearly about 2,100 newly described species are added to WoRMS. Over the years, the Data Management Team has noted that editors sometimes lag 1–2 years behind in adding these species, which explains the lower figure for 2016 (Fig 3B).

## Solving mysteries through cross-taxa collaborations between editors

The documentation of some taxa has posed problems, as their names were–correctly or incorrectly–in use in different taxonomic groups, sometimes with information dating back to pre-Linnean times. Correctly documenting such taxa in WoRMS has only been possible through the collaboration of editors across taxa and by supporting them to find solutions. Some notable cases in WoRMS include the genera *Scolopendra* and *Alcyonium*. Issues around the genus *Scolopendra* arose when the Data Management Team bulk-imported a number of Myriapoda into WoRMS as part of a data-rescue action, including species under the genus *Scolopendra*. As the *Scolopendra* genus was already available under Polychaeta, the new myriapod *Scolopendra* species were added to this already existing genus. The WoRMS chief Polychaeta expert pointed this out as an error, thereby initiating an extensive search and long, constructive discussion between editors of both the Myriapoda and the Polychaeta on how to deal with this situation. The name *Scolopendra* was already in use in the time of Aristotle [12] and back then it referred to both land and sea animals which were somewhat alike, including myriapods and polychaetes [13 and references therein]. *Scolopendra marina* was originally documented under the Polychaeta in WoRMS, as this name was used in pre-Linnaean times for nereid-like worms, and in use by many authors (see [14]). A search on the Biodiversity Heritage Library [15] showed that the first mention of *Scolopendra marina* dated back to 1531. Other *Scolopendra* species added to this genus through the bulk-import were, however, not all Polychaetes, complicating the situation and requiring a solution. On top of that, there was an issue with the documented authority of *S*. *marina*–Linnaeus, 1758 versus Slabber, 1781 –which also needed to be clarified. Literature research by the involved experts indicated that the genus *Scolopendra* belongs to Myriapoda, that *S*. *marina* is currently considered a nomen dubium [16], and that the correct authority of *S*. *marina* is Linnaeus, 1758 [17–19]. It was however recognized that users of the Polychaeta portal should still be able to easily retrieve information on this species, thus–through the use of contexts–it is also assigned to the Polychaeta portal. In general agreement, the responsible Polychaete editor can now also edit the *Scolopendra* genus within the Myriapoda, as this taxon bears relevance for his group and relevant notes have been added to clarify the situation [16,17].

The *Alcyonium* case shows similarities with the *Scolopendra* case, in the fact that the name *Alcyonium* also dates back to the ancient Greeks, and was used by pre-Linnaean authors for various groups of unrelated marine sessile organisms, including Octocorallia, Porifera and Tunicata (e.g. [20]). Linnaeus himself has formally named *Alcyonium* and listed three species, belonging to different groups [18]. Later on, other authors have also named *Alcyonium* species, also belonging to various groups, such as Tunicata (e.g. [21–23]), Porifera (e.g. [24,25]), and Hydrozoa (e.g. [26]). Within the WoRMS database, several *Alcyonium* genera had been added with unofficially assigned—*sensu*—authorships to distinguish between the use of *Alcyonium* in the Porifera, Cnidaria and Tunicata. This way, each group could manage the *Alcyonium* names relevant for their group. Although this approach seemed to work from a database point of view, it was incorrect and violated nomenclatural rules. The issue was raised recently by the chief taxonomic editor of the Porifera, urging to merge all *Alcyonium* species under its rightfully classified genus, *Alcyonium* Linnaeus, 1758 in the Octocorallia [27] and to strive for shared editing rights for this genus among the editors of the involved groups. This suggestion was followed and the present situation now allows the Porifera and Tunicata editors to document their group-specific *Alcyonium* species, avoiding duplication and confusion within WoRMS. Each of the originally wrongly classified taxa is now fully documented in WoRMS and now point to the currently accepted name, within the correct classification (e.g. [28–32]).

Although these situations require a lot of time and research to sort out and the effort behind it mostly remain invisible, the benefit is that complicated cross-taxa cases are thoroughly being documented in WoRMS [16,17,27], and create clarity for all future users and editors.

In total, a little over 11,000 species fall under shared custodianship of editors responsible for different taxa.

## WoRMS usage

### Through the website, web services and downloads

**Fig 4:**
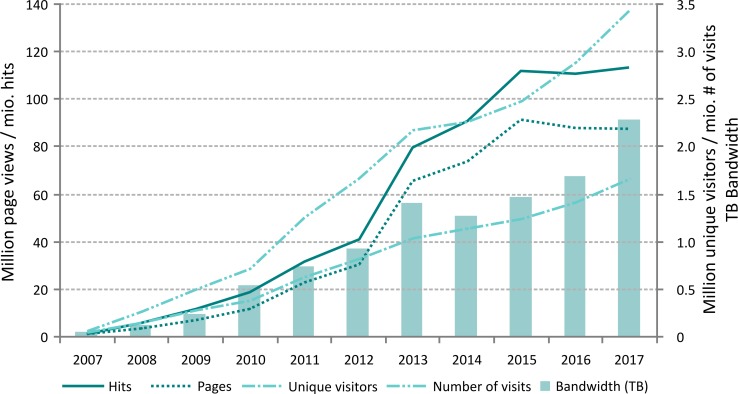
Compilation graph with the evolution in page views, hits, unique visitors, visits and terabytes of bandwidth for the website of the World Register of Marine Species.

The WoRMS portal has been up and running since 2007, with a steady growth in unique visitors and hits (Fig 4). Currently, there are on average 4,500 unique visitors per day and about 4.6 million hits to a marinespecies.org related portal per month. At the time of writing, 65 institutions or data systems from 28 countries are making use of one or more of the 16 available WoRMS web services and/or they provide deep links to WoRMS. Although this is already a significant amount, this is likely an underestimation, as the DMT cannot automatically track the usage of the offered services. The list is being maintained based on the fact that people inform the DMT on their usage of one or more of the available services. The list of known, registered users is available online at http://www.marinespecies.org/users.php.

The three most popular web services are (1) Get AphiaID, enabling the retrieval of the AphiaID for your taxon, (2) GetAphiaRecordByID enabling the retrieval of the available taxon information for a specific AphiaID and (3) getAphiaRecords enabling the retrieval of all possible options–e.g. homonyms or similarly spelled names. This last service was made available in September 2013.

**Fig 5:**
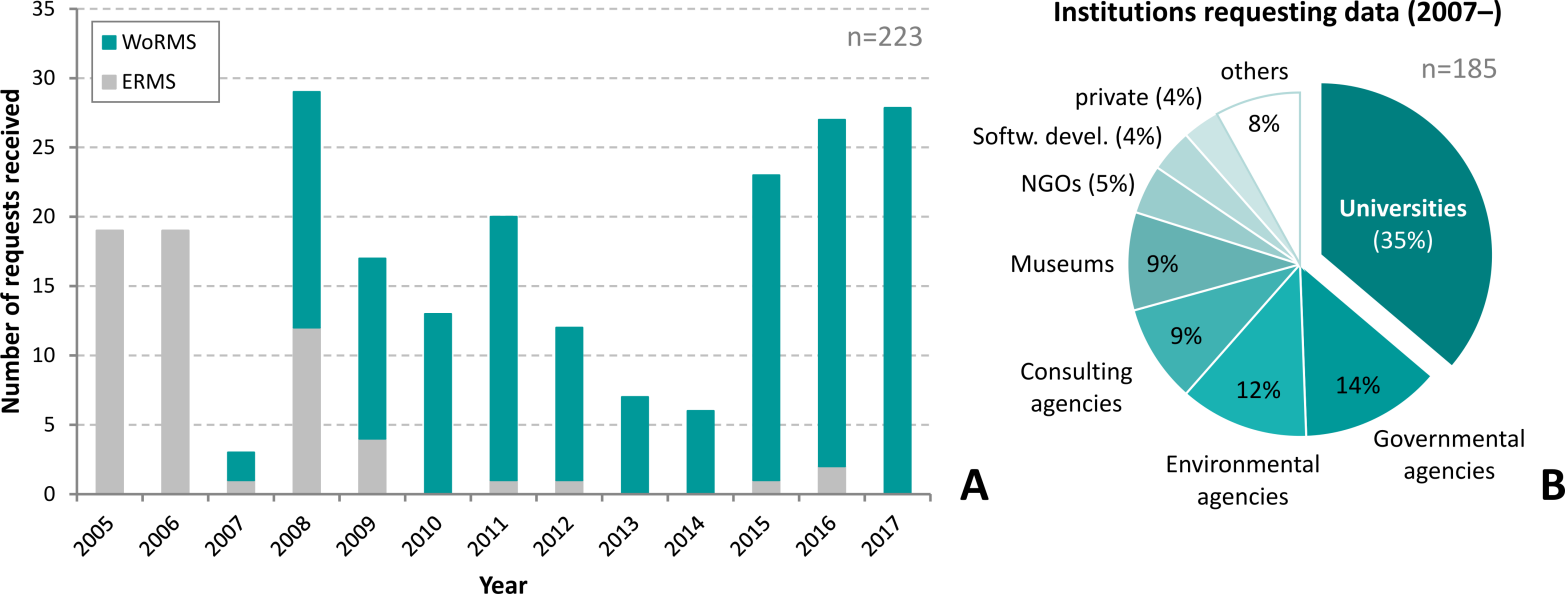
Temporal and institutional distribution of data requests received. A: stacked bar chart showing number of data request per year starting in 2005. Requests for ERMS data are shown grey colour, those for WoRMS data in turquoise.; B: pie chart shows in terms of percentage which type of organisations request WoRMS downloads.

While the majority of WoRMS’ users access the database content via the WoRMS website (manually) or through the offered web services (see above) in an automated fashion, some users have more specific needs and require data dumps. WoRMS offers such downloads upon request and provides monthly updates of these data dumps for registered users once the request has been approved. Users requesting WoRMS data as downloads must fill in the Data Request form (available on the WoRMS website: http://www.marinespecies.org/usersrequest.php) and provide details on intended usage and user details. Applications are screened and approved by designated members of the WoRMS SC in collaboration with the WoRMS DMT. Criteria for evaluation of the application include whether the applicant(s) intend to re-distribute WoRMS data or to use them for internal purposes. Typically, several requests are received per month (Fig 5), which are processed within a few days. Access is granted for a period of one year at a time and requests for applications that necessitate access over longer periods need to be renewed annually. Currently, 119 organisations or data systems from 35 countries are using WoRMS by accessing the monthly downloadable versions.

**Fig 6:**
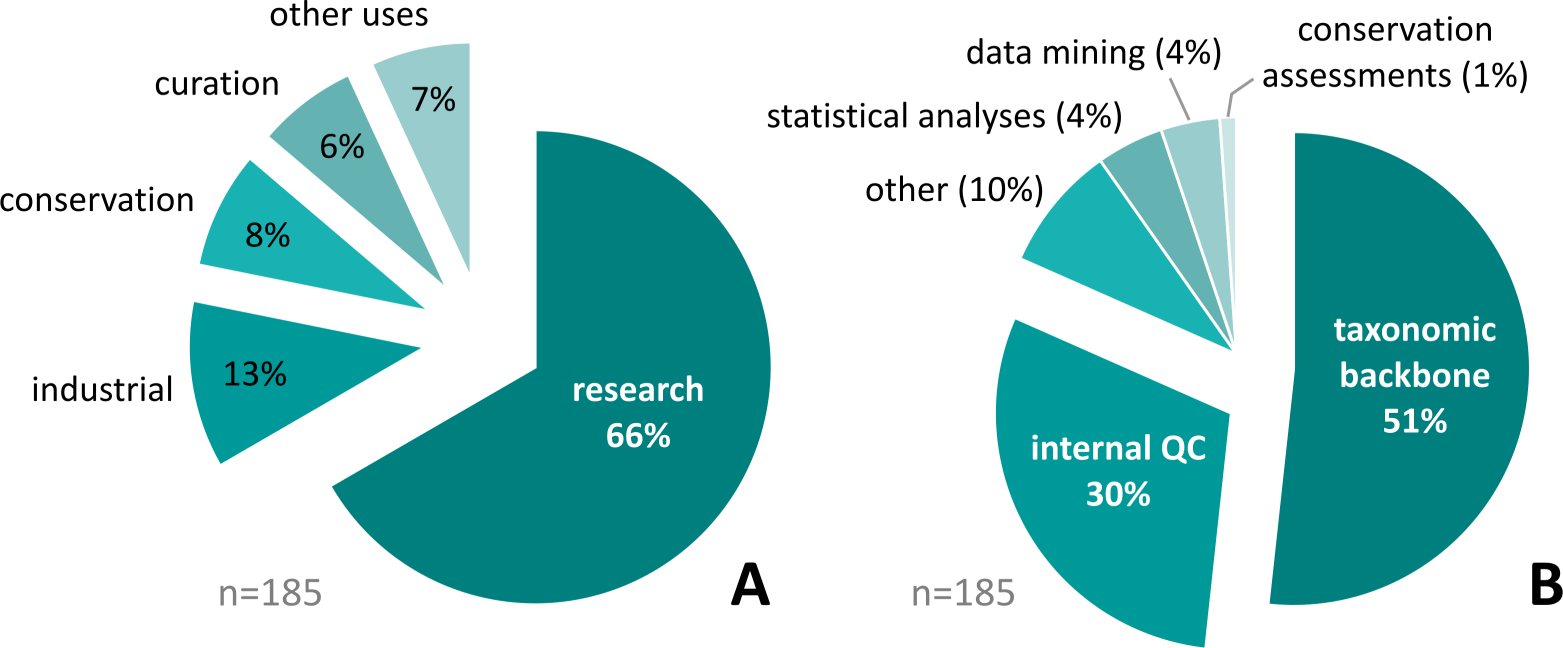
Planned usage (A) and application (B) of WoRMS data by data request applicants since 2007 showing that the majority of use cases are relate to scientific research where WoRMS data either forms the taxonomic backbone of research databases or a tool for data quality control.

Persons requesting WoRMS downloads range from individual researchers to representatives of national and international agencies (e.g. ICES), including NGOs, commercial companies, and governmental bodies. The large majority of these use WoRMS data as taxonomic backbone and/or for quality control of their internal databases (Fig 6B), improving data consistency, enabling continuous automated taxonomic updates, and cross-institutional data exchange by reference to a persistent, globally unique identifier (Life Science Identifier—LSID). Subsidiary uses include usage of WoRMS content as a thesaurus for data mining, as a basis for conservation assessments, and a variety of statistical analyses.

WoRMS content has been used within a wide variety of different projects (e.g. some NSF Tree of Life projects, the Moorea Biocode Project) and for improving and enriching the content of a numerous databases, ranging from institutional (such as Senkenberg’s *SeSAM* inventory database), to national (*e*.*g*., the *L'Inventaire national du Patrimoine naturel* in France, or the *Greek Taxon Information System*), and international initiatives (*e*.*g*. OBIS, Global Names Index, OpenTree of Life, Map of Life, SeaLifeBase and SeamountsOnline). Even specialized databases for genetic resources with thousands of views per day make use of WoRMS data for quality control of their taxonomies (*e*.*g*., BOLD and the NCBI taxonomy database, which forms the taxonomic backbone of GenBank).

**Fig 7:**
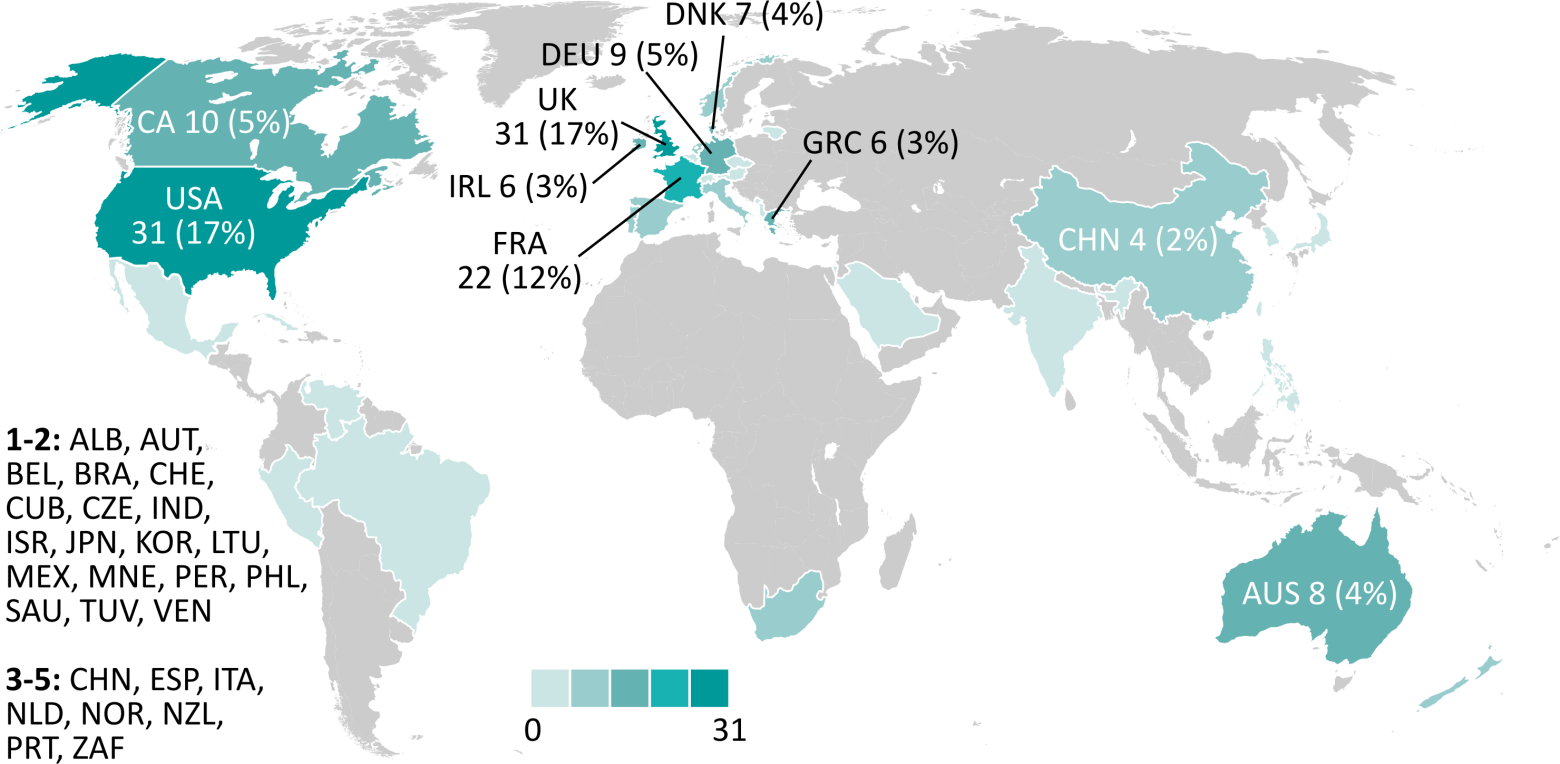
Usage of WoRMS data downloads by different nations: number of requests received since 2007 and percentage of total requests since 2007 (n = 185). Country abbreviations following the ISO 3166–1 alpha-3 code. Map based on https://commons.wikimedia.org/wiki/File:BlankMap-WorldSICN.svg created by Gap5200 and released under a CC BY-SA 4.0 license.

Geographically the large majority of data requests were received from projects and institutions located in Europe (59% of the requests since the launch of WoRMS in 2007; Fig 7), followed by North American ones (23%). The countries making most use of the option to download data were the UK and the US, with (17% each), followed by France (12%; n = 22). This may in part be related to governmental investment in marine research in these countries, as well as the geographic location of international projects and institutions drawing on WoRMS data.

Users requesting the data include academic publishers, national academies of sciences, advertising agencies, dive clubs, individual researchers, marine stations, NGOs, oil companies, software developers, and aggregators of taxonomic data. Power users of the data, however, are universities (35% of the requests), followed by various governmental offices (14%), and environmental agencies (12%). Consulting companies and natural history museums account for the remaining requests (9% each) (Fig 5B).

Usage of WoRMS data as a taxonomic backbone for internal and public databases prevails as the top reason stated in the requests for access (51%), followed by quality control of existing databases (30%). Most of these are related to scientific research (66%), followed by industrial applications (13%), conservation efforts (8%), and museum collection databases (6%), illustrating the strong and varied impact WoRMS has on matters relating to the use and classification of and research on marine organisms (Fig 6A).

### Through taxon match tool

**Fig 8:**
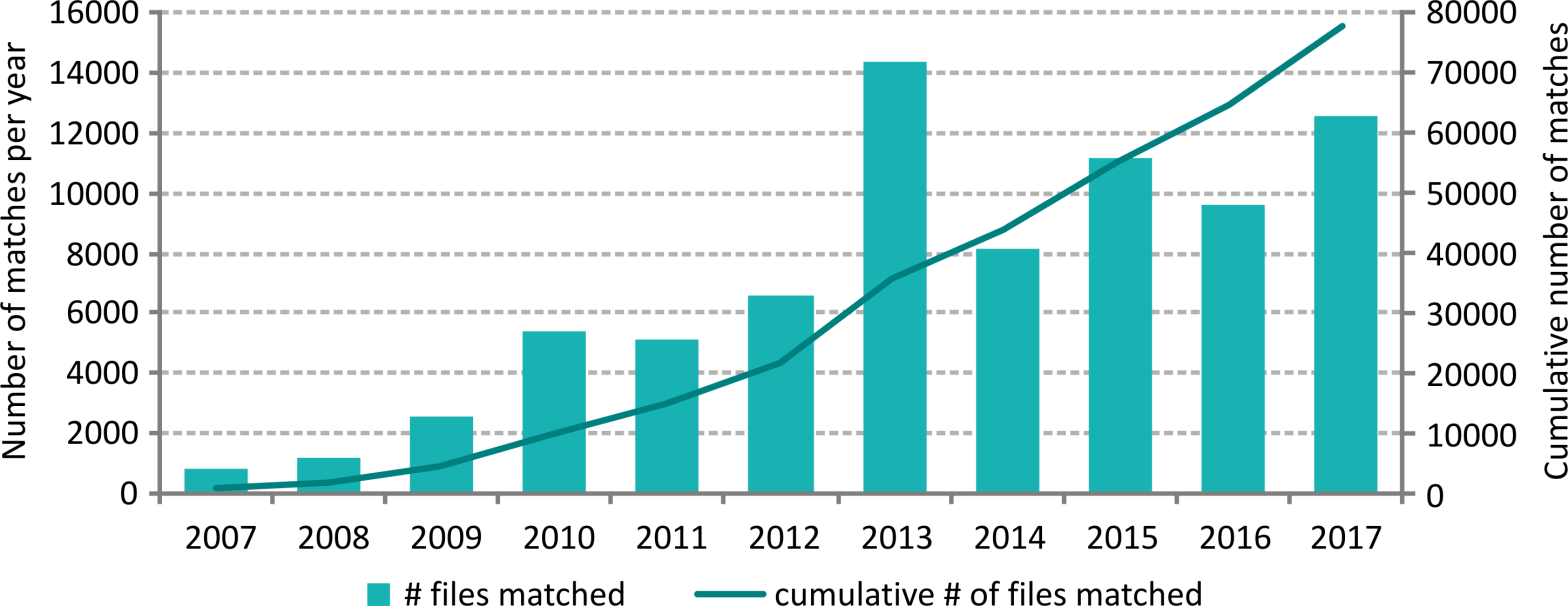
The absolute number of files matched per year on the left Y-axis, and cumulative number of files matched through the WoRMS taxon match tool since its launch in 2007 (right Y-axis).

The Taxon Match Tool on WoRMS is a freely accessible service where users can automatically match their own taxon list with the World Register of Marine Species [33]. It is based on a fuzzy matching approach, first developed by Tony Rees [34–35–36]. Since it was put into operation for WoRMS, more than 65,000 files have been uploaded and matched, with an average of 7,000 files per year (Fig 8).

Following the success of the taxon match tool offered through the WoRMS website, it was decided to also make this tool available through the data services of LifeWatch Belgium (www.lifewatch.be/data-services), offering further possibilities: (1) get AphiaID from WoRMS, (2) taxon match with WoRMS, (3) reverse WoRMS taxon match by AphiaID and (2) taxon match with a selection of 7 of the WoRMS regional and thematic sub-registers. These services allow users to retrieve specific information from WoRMS and related sub-registers, based on either a scientific name or an AphiaID they send through the services. A user can also combine any of these services, thereby checking whether some or all of the taxa in his list are part of a specific region or are considered to be e.g. deep-sea or introduced species. These LifeWatch services have been available since 2013, with continuous growth in usage. The mentioned services had been used almost 1.000 times (statistics retrieved on July 31^st^, 2017). Next to the more elaborate taxon matching options through LifeWatch–compared to the general Taxon Match on WoRMS–these taxon-matching services can be combined with other available services at LifeWatch, including geographically-oriented services, e.g. linked to the Ocean Biogeographic Information System–OBIS [37] or Marine Regions [38] to retrieve documented occurrences of species and link these to e.g. Exclusive Economic Zones or other geographical subdivisions.

### In scientific literature

**Fig 9:**
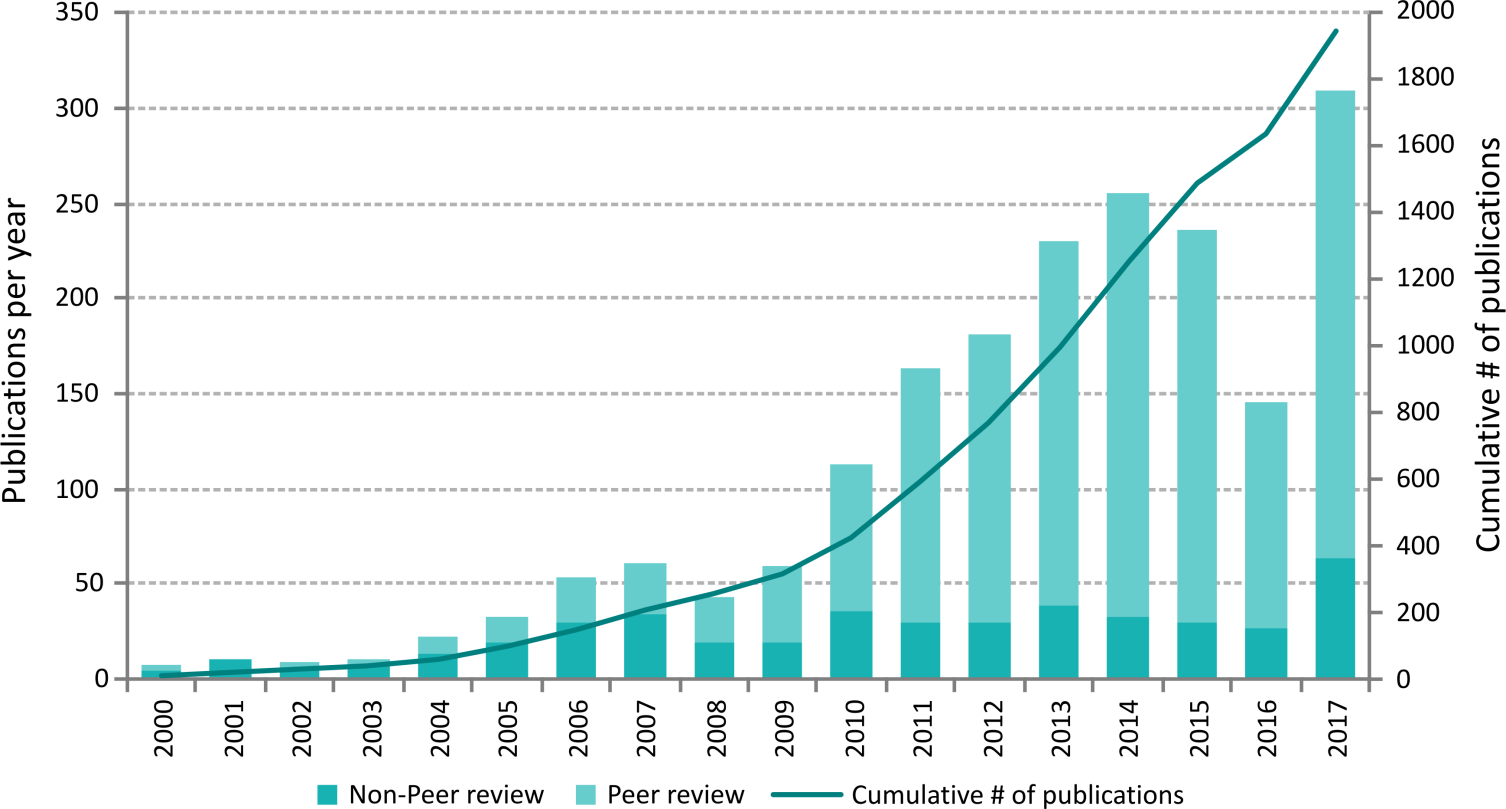
On the left Y-axis: the number of peer-reviewed and non-peer-reviewed publications that refer to WoRMS in a given year. Pre-2008 publications are publications that refer to one of the registers preceding WoRMS. Right Y-axis: cumulative number of publications (peer-reviewed and non-peer-reviewed that refer to WoRMS).

The library at the Flanders Marine Institute (VLIZ) keeps track of all publications that refer to or mention WoRMS or any of its sub-registers in their abstract, full text or references. To compile this list, several resources have been consulted including Web of Science, SCOPUS and Google Scholar. For each publication, it was carefully checked whether WoRMS or any of its sub-registers was mentioned, based on a predefined list of keywords. Early 2018, 1496 peer-reviewed and 447 non-peer-reviewed publications have been identified as citing or mentioning WoRMS (Fig 9).

In the early years after the launch of WoRMS there were slightly more references to the database in non-peer-review publications, such as newsletters, reports and conference abstracts. 2010 seems to be a pivotal year where the mention and use of WoRMS in peer-reviewed scientific literature increased, while mentions in non-peer-reviewed literature remained more or less steady over the last decade. This increase in citation in the scientific literature indicates the reliance that many disciplines have on a stable taxonomic classification and up-to-date list of marine species.

### Through other data systems

In the past decade, other data systems have requested access to the taxonomic content of the World Register of Marine Species (WoRMS), with the goal to integrate this into their own online system, thus redistributing WoRMS content. For these requests, dedicated memoranda of understanding (MoUs) have been set up, clearly describing the collaboration between WoRMS and the other party, and always striving towards a bilateral cooperation and data-exchange. There are currently 5 instances receiving (part of the) WoRMS content through such an MoU, amongst which the Catalogue of Life (CoL) [39] and the Encyclopedia of Life (EoL) [40].

**Fig 10:**
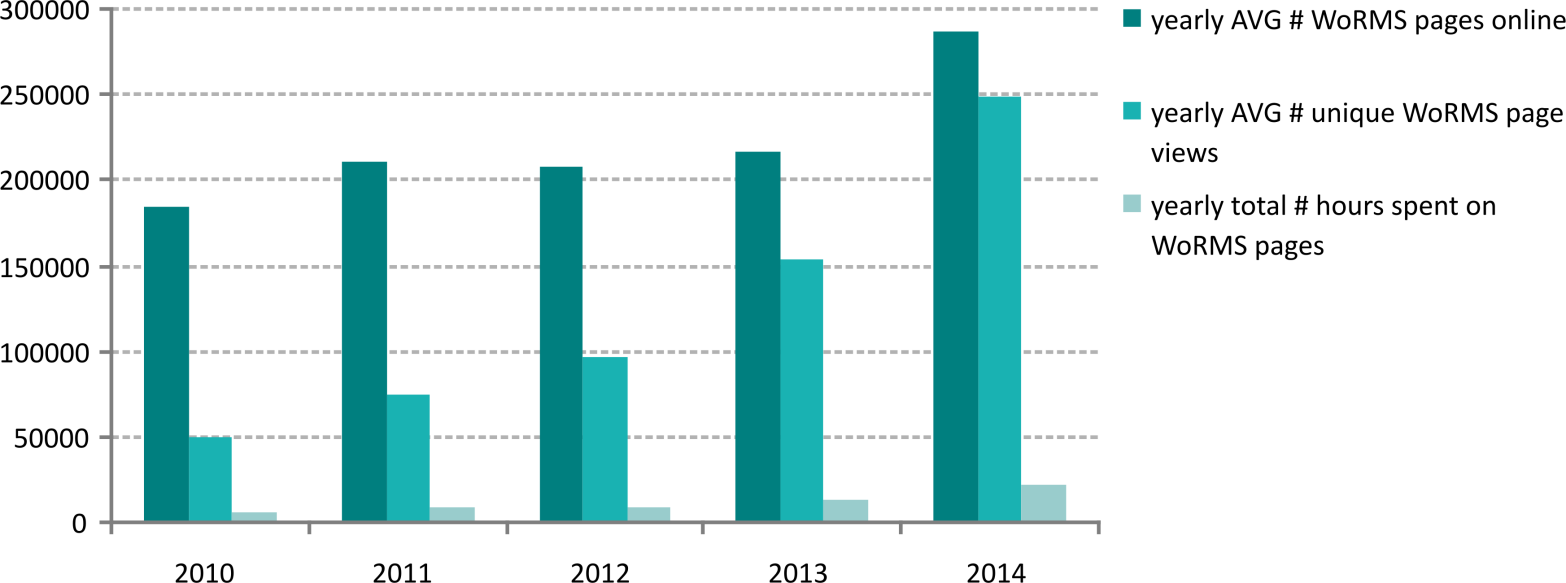
An analysis of the monthly web stats on the WoRMS provided pages to Encyclopedia of Life (EoL), based on the EoL web stats tool for data providers (date of analysis: 3 August 2017).

These systems, however, do not always keep track of the usage of the WoRMS information that is provided. Only (partial) statistics could be retrieved for the Encyclopedia of Life. On average, 6.8% of the online EoL pages come from WoRMS. An analysis of the available monthly web statistics from 2010 to 2014 (www.eol.org) (Fig 10) shows that the use of WoRMS-related pages available through EoL has increased tremendously: in 2010, only 26% of the WoRMS-related pages accessible through EoL were viewed, increasing to 86% in 2014. Over the 5 years analyzed (2010–2014), EoL users have spent 11,650 hours on pages with information provided by WoRMS, with a slightly increasing trend. This corresponds to an average more than 20% of the time users spend on EoL pages in general.

### Through “clones”

A fair amount of information on marine taxa on Wikipedia links back to WoRMS. At the time of writing (3/8/2017), 199,000 Wikipedia pages carry a reference to WoRMS, supplemented by about 15,500 WikiSpecies pages referring to WoRMS. Some of these wiki-pages–e.g. on Mysidae [41]–include the full classification and an overview of described genera and species from WoRMS. Although it is noteworthy that an initiative like Wikipedia makes use of an online available resource such as WoRMS, the information seems to be retrieved once, and is not further updated within the Wiki-pages, quickly leading to outdated online information on the pages of this highly used and well-known internet resource.

As long as such “cloning” initiatives properly credit and refer to WoRMS, and under the condition that it is clear that the Register itself contains the most up-to-date information, these actions are welcomed as they help raise awareness of the existence of WoRMS, although it is clear that improved methods of tracking updates should be explored. On several Wikipedia and WikiSpecies pages on larger taxonomic groups, WoRMS is also included under the (external) links section (e.g. Mollusca, Cumacea, Porifera, Polychaeta, …) and is therefore clearly a much used source of information for creating Wiki-pages.

## User feedback & questions

The strength and impact of the WoRMS user community cannot be underestimated (see also [4]): they detect omissions overlooked by our editors, possible errors or duplicate entries and they inform the Data Management Team (DMT) on this through info@marinespecies.org. The DMT acts as a buffer between the many users and editors, to avoid editors being overloaded by questions and remarks that–in many cases–can also be solved by a non-expert, e.g. looking up and adding original descriptions of taxa or correcting obvious spelling errors in the names and dates of authorities. Questions that do need expert input–e.g. on homonyms or unclear synonymies or missing taxa–are forwarded to the relevant experts, who either correct or add information to WoRMS, or provide appropriate feedback to the users. Users also have an eye for discovering (possible) duplicates in the database and the correction or addition of the year of publication of a taxon, as their specific searches sometimes unravel these more easily compared to standard quality control checks run by both the DMT and editors. The user feedback is a valuable asset in making WoRMS more complete and keeping the already available data up-to-date. Questions commonly received by the DMT deal with species identifications on photographs, taken during diving and snorkeling trips or of (partial) animals found washed ashore. In these cases, the DMT tries to avoid sending these to the editors–due to the large amount of such requests received from some groups–and points people to other relevant sources and instances that might be able to help them with an identification. Cases where images were actually forwarded to editors for identification, showed that these images commonly lacked particular details needed for identification.

Since taking the info@marinespecies.org email into use in August 2007, almost 148,000 emails have been received. In 2012, the annual number of emails surpassed 10,000 and since 2015, the Data Management Team is dealing with more than 20,000 emails annually. These emails include all communication between the DMT, the Steering Committee and the Editorial Board, notifications of actions taken by editors, user questions and feedback, questions from editors regarding the use of the online edit interface or requests for support in uploading large amounts of data and information, and a small amount of junk email.

## Connecting editors: How to keep a worldwide network of experts interactive, motivated and on track

Keeping in touch with the full network of editors is important in order to ensure WoRMS remains the most up-to-date resource of its kind, especially given the size of both the system and the editorial board. Email is the most used tool by both the DMT and the SC to inform the involved experts on news, decisions, and changes. In addition, parts of the editorial board are brought together–formally or informally–on several occasions, giving them and the DMT a chance to discuss issues at hand or tackle specific gaps in WoRMS. This can be by organizing small, informal meetings at conferences with the present editors or through dedicated meetings, workshops, and data grants.

A first WoRMS taxonomic editor workshop was organized in Oostende, on 20–21 June 2008, bringing together 55 marine scientists from 17 countries. On this occasion, WoRMS was officially inaugurated [42,43]. In later years, the Data Management Team has aimed to informally bring editors together during the World Conferences on Marine Biodiversity (WCMB), organized with intervals of 3 to 4 years. These occasions are used to briefly present the state of the art on WoRMS, and to listen to suggestions and ideas of the editor community.

Through the LifeWatch project, VLIZ has been able to use LifeWatch funds to organize dedicated editor workshops, aimed at bringing together taxonomic, thematic or regional editorial groups and allowing them to better organize themselves, to discuss pending issues and to make short- and long-term plans for the future. Each workshop has included a detailed demonstration of the online interface and available tools on WoRMS. Since 2014, ten such workshops have been sponsored, bringing together 111 taxonomic and/or thematic experts. The organization of such workshops has proven very successful: the involved editors have met and are mostly highly motivated to keep the momentum going and to strive for the accomplishment of the pre-set goals. Even 1 or 2 years after such a workshop, the experts remain active and closely collaborate with their colleagues and the DMT, thereby facilitating further progress and dealing with possible user questions and issues. Although it takes effort to organize such workshops, it is a worthwhile investment and has proven its value in making WoRMS more complete in a very dynamic way.

Gaps in WoRMS have also been specifically targeted by assigning data grants to the editors or scientists responsible. A first series of data grants was initiated in 2009 through the Census of Marine Life, targeting specific gaps in marine Mollusca and marine parasite species belonging to different groups. Between 2012 and 2015, LifeWatch was able to support 41 data grants, all targeted at improving the content and quality of WoRMS and making sure that the information already available is validated. These LifeWatch grants led to the addition of more than 10,000 missing species to WoRMS. In contrast to workshops, the work through data grants moves forward faster: the work linked to a data grant is mostly carried out within 2–6 months and thus quickly contributes improvements to WoRMS. The DMT has, however, noticed that experts do not necessarily stay motivated to keep the momentum after the grant has been completed. Taking into account this experience, data grants seem to be a good approach to tackle small remaining gaps, while organizing workshops–with a dedicated training in the use of the online interface–is more beneficial in the long run, as it keeps a larger number of experts involved and motivated to contribute to WoRMS.

As the estimated target of 230,000 marine species was reached in 2015, the Steering Committee has now produced a list of priorities for editors to tackle in order to make WoRMS more complete with high quality, relevant, taxon-related information. These priorities include the documentation of the original name, to complete the authorship, to document the original description of each available species, and to document type localities for each species and type species for each genus. Each editor–with support of the DMT–is now putting effort in dealing with these priorities, in combination with the addition of missing names and newly described species within their group of expertise.

## WoRMS–part of a bigger picture

The World Register of Marine Species is unique. No comparable global database for marine species exists, which is driven by an expert community, continuously supported by a Data Management Team and which can rely on a permanent host institute, dedicated to keep the Register online.

Several global initiatives have sought collaboration with WoRMS, as an expert provider of taxonomy to their own data systems. Since 2009, WoRMS has been sharing Global Species Databases with the Catalogue of Life (CoL) [39]. In 2017, the number of provided GSDs mounted to 55, representing more than 145,000 species. Each GSD provided by WoRMS is automatically updated in CoL on a monthly basis and due credit is given to the involved editors. Whether a GSD is delivered to CoL or not, depends on the needs of CoL and the available source databases. As they work with multiple providers, they analyze the offered content of these providers, making decisions based on completeness and how up-to-date each list is.

The content of WoRMS is also shared with the Encyclopedia of Life (EoL) [40] since 2014. Here, all taxon names (accepted & synonyms) are delivered to EoL on a monthly basis, together with information on the higher classification, the documented distributions and a selection of the available notes. A monthly copy of WoRMS is also in sent to the Global Biodiversity Information Facility (GBIF) [43], where it is incorporated in their overall taxonomic backbone, and used to check the accuracy of the names in the GBIF data system.

WoRMS also shares dedicated content with the database of the Freshwater Animal Diversity Assessment (FADA) [44,45]. As some taxonomic groups have representatives in both the marine and the freshwater environment (e.g. Mollusca and Amphipoda), meetings between WoRMS and FADA editors of these overlapping groups have led to avoidance of effort duplication, by managing a specific group in only one system. If the majority of the group are marine species, then the responsible editor can decide to also manage the freshwater species within the Aphia database [the platform behind WoRMS and other related databases [4], and these can be sent to FADA on a regular basis.

The content of WoRMS [4] also contributes to the LifeWatch Taxonomic Backbone (LW-TaxBB), initiated in 2012. This Taxonomic Backbone is developed and managed at the Flanders Marine Institute, the host institute of WoRMS and contributes to the European level of the LifeWatch project. LifeWatch is a European E-Science Infrastructure for biodiversity and ecosystem research, a distributed virtual laboratory which is used for different aspects of biodiversity research. LifeWatch needs species information services for the standardization of species data and the integration of the distributed biodiversity data repositories and operating facilities. All these services together compile the LifeWatch Taxonomic Backbone and–on a taxonomic level–contain taxonomy access services and a taxonomic editing environment identical to the one used in WoRMS. The LW-TaxBB needs to provide species names, their higher classification, literature references linked to the available information, images, morphology descriptions, information on habitat, ecological traits, distributions, and occurrence data. Although the Taxonomic Backbone does not solely focus on marine species, WoRMS is at this moment the largest contributor to the LW-TaxBB and the WoRMS community is thus significantly contributing to the success of LifeWatch.

## Moving forward

Keeping the existing momentum in WoRMS is essential. The last ten years have proven that WoRMS is following a successful approach, through its governance and its open, dynamic and easily accessible data and information both through the website and web services. The WoRMS SC plays an important role in mobilizing taxonomic and thematic editors willing to voluntarily contribute to WoRMS and to put forward priorities that are achievable and supported by the full community. The support of a dynamic and accessible Data Management Team in all this is invaluable, and its continuity is essential in the future development and success of WoRMS.

Since 2012, the Data Management Team (DMT) of WoRMS has been financially supported through LifeWatch Belgium. LifeWatch also secured funding for the highly successful data grants and editor workshops. Through such support (see earlier), editors have been able to address long-standing gaps in the content of WoRMS (e.g., Digenea, Gastrotricha & Tardigrada). Prior to the launch of WoRMS, the European MarBEF Network of Excellence project also allocated funding to fill major gaps in the world inventory through dedicated data grants, giving an enormous boost to the content of the European Register of Marine Species (ERMS), the predecessor of WoRMS. It is hoped that funding can be maintained for the next 10–20 years through LifeWatch and other resources, as this support has facilitated numerous possibilities for the SC, the DMT, and the Editorial Board to take action to make WoRMS more complete and to develop new tools and functionalities in order to allow easy access to the available data for science and society.

In its 10 years existence, WoRMS has become more and more embedded in other international initiatives, either as a resource for high-standard taxonomic information, as a possible platform to store taxon-related information, or as part of bilateral collaborations to avoid duplication of efforts. Recently, WoRMS has been approached by the SeaLifeBase initiative to discuss the possibilities of a bidirectional data exchange, allowing each system to invest in its own strengths and to benefit from the others’ efforts. Such collaborations should be aspired to, thereby making sure that existing (international) initiatives and available expertise are interlinked and that duplication of effort is avoided. Although the aims of initiatives and projects might differ, there is very often a common ground upon which a collaboration can begin.

On 6 November 2017, the WoRMS website received a full make-over, ensuring that it keeps track with modern technological advances, both in look-and-feel, and in order to facilitate improved functionality. Together with its new look-and-feel, WoRMS has left its’ childhood behind and is ready to serve the marine scientific community for the next decades to come.
